# Traditional Chinese medicine suppresses left ventricular hypertrophy by targeting extracellular signal-regulated kinases signaling pathway in spontaneously hypertensive rats

**DOI:** 10.1038/srep42965

**Published:** 2017-02-22

**Authors:** Xingjiang Xiong, Xiaochen Yang, Lian Duan, Wei Liu, Yun Zhang, Yongmei Liu, Pengqian Wang, Shengjie Li, Xiaoke Li

**Affiliations:** 1 Department of Cardiology, Guang’anmen Hospital, China Academy of Chinese Medical Sciences, Beijing, China; 2 Department of Cardiology, Beijing Hospital of Traditional Chinese Medicine, Capital Medical University, Beijing, China; 3 Department of Molecular Biology, Guang’anmen Hospital, China Academy of Chinese Medical Sciences, Beijing, China; 4 Department of Pharmacology, Institute of Chinese Materia Medica, China Academy of Chinese Medical Sciences, Beijing, China; 5 Department of Molecular Biology, Peking Union Medical College Hospital, Chinese Academy of Medical Sciences, Beijing, China; 6 Bio-organic and Natural Products Laboratory, McLean Hospital, Harvard Medical School, Belmont, USA

## Abstract

Chinese herbal medicine Bu-Shen-Jiang-Ya decoction (BSJYD) is reported to be beneficial for hypertension. Over expression of extracellular signal regulated kinases (ERK) pathway plays an important role in left ventricular hypertrophy (LVH). This study aimed to observe effects of BSJYD on LVH in spontaneously hypertensive rats (SHRs) and explore its possible mechanism on regulation of ERK pathway. Sixty 12-week-old SHRs were randomly allocated into 5 groups: BSJYD high dose group, middle dose group, low dose group, captopril group, and control group. Besides, a control group of Wistar-Kyoto rats was established. All rats were treated for 8 weeks. Systolic blood pressure (SBP), heart rate (HR), pathology, and left ventricular mass index (LVMI) were measured. Western blotting and Real-time PCR were used to assess the expressions of BDNF, Ras, ERK1/2, and c-fox levels. SBP and HR were significantly decreased compared with the control group and LVMI was markedly improved by BSJYD treatment in a dose-dependent manner. BSJYD inhibited the expression of BDNF, Ras, ERK1/2, and c-fox mRNA in LVH. In conclusion, BSJYD suppressed hypertension-induced cardiac hypertrophy by inhibiting the expression of ERK pathway. These changes in gene expression may be a possible mechanism by which BSJYD provides myocardial protection from hypertension.

Hypertension is an important worldwide public health challenge because of its high prevalence and concomitant increase in risks of cardiovascular, cerebrovascular and renal diseases^1^,^2^. It has been identified as the leading risk factor for mortality in human populations, and is ranked third as a cause of disability-adjusted life-years^3^,^4^. Hypertension is not only manifested by an elevated arterial blood pressure (BP), but also involves complex structural and functional alterations of its target organs^5^. In the hypertensive state, a number of adaptive changes occur in ventricle. Cardiac hypertrophy results from increased mechanical load on the heart and through the action of neurohumoral mediators. Left ventricular hypertrophy (LVH) is a cardinal manifestation of hypertensive organ damage associated with an increased cardiovascular morbidity and mortality^6^,^7^. LVH in hypertensive patients may be regarded as a powerful, independent biomarker reflecting the impact of pressure overload as well as of several risk factors on heart^8^. These structural abnormalities may play an important role in the development and maintenance of hypertension, because they may eventually lead to decompensation and ventricular extension, thus increasing the risk of heart failure and sudden death. Studies have confirmed that, with the development of LVH, the incidence of cardiovascular events has increased by 6–10 times^9^,^10^,^11^,^12^. Therefore, suppression of LVH was shown to improve cardiovascular outcome independently of other risk factors, and thus has been suggested as an intermediate endpoint^13^,^14^.

The mitogen-activated protein kinases (MAPKs) have been implicated as focal mediators of cardiac hypertrophy in both cell culture and genetically modified mouse models^15^. The extracellular signal-regulated kinase (ERK) signaling pathway, a branch of the greater MAPK signaling cascade, appears to induce a unique form of concentric cardiac hypertrophy^16^. It is activated in response to almost every stress- and agonist-induced hypertrophic stimulus examined to date, suggesting the straightforward hypothesis that these kinases are required for promoting the cardiac growth response^17^. Signaling through ERK cascade is classically initiated at the cell membrane by activation of the small G protein Ras that then recruits the MAP3K Raf-1 to the plasma membrane, where it is activated^18^. More and more evidences showed that over expression of ERK signaling pathway plays an important role in cardiac hypertrophy^19^,^20^,^21^.

Traditional Chinese medicine (TCM) has been reported to be effective for the treatment of hypertension^22^,^23^,^24^. However, there was little information available in literature about whether Chinese herbal medicine with anti-hypertensive effect could affect ERK pathway in LVH. Herbal compounds Bu-Shen-Jiang-Ya decoction (BSJYD) has been widely used in treating hypertension with kidney yin deficiency syndrome for many years. In our previous study, a prospective case series involving 108 hypertensive patients with kidney yin deficiency syndrome revealed a significant reduction of 13.1 mmHg of systolic blood pressure (SBP) and 9.30 mmHg of diastolic blood pressure (DBP) by BSJYD^25^. Additionally, no acute toxicology was identified on mice^26^. The significant BP-lowering effect of BSJYD has been confirmed in spontaneously hypertensive rats (SHRs); moreover, the possible cardioprotective mechanisms of BSJYD on hypertension may be related to up-regulating adiponectin and improving insulin resistance^27^. However, further study of its detailed anti-hypertensive and reversing ventricular hypertrophy mechanism is still needed. Here, we aimed to examine how ERK signal transduction induces LVH and whether BSJYD inhibits LVH bioactivities through the ERK signaling pathway effectively.

## Results

### Effect of BSJYD on SBP

After treatment for 8 weeks, SBP was significantly decreased in the Ca group, the Bh group, the Bm group, and the Bl group compared with the C group (*P* < 0.05). Compared with the Ca group, SBP was significantly increased in the Bm group, the Bl group, and the C group (*P* < 0.05), while no significant differences were identified between the Ca group and Bh group (*P* > 0.05) (as shown in Table 1).

### Effect of BSJYD on HR

As shown in Table 2, HR were significantly lowered after 8 weeks in the Bh group (*P* < 0.01), while HR in the Bm group, the Bl group, and the Ca group were not significantly lowered when compared to the C group. As compared with the Ca group, the Bh group was significantly lowered (*P* < 0.05), while there is no significantly difference in the other groups (*P* > 0.05).

### Effect of BSJYD on LVMI

**Figure 1:**
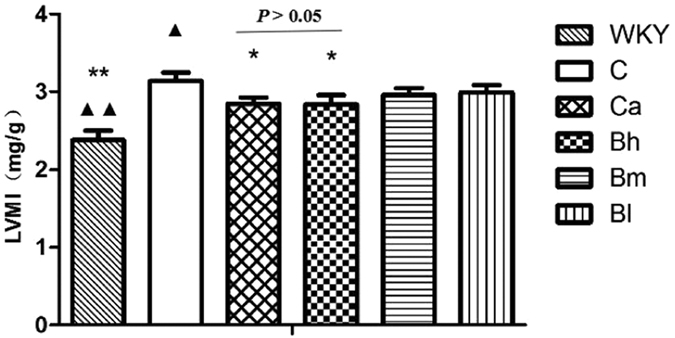
Effect of Bu-Shen-Jiang-Ya decoction on left ventricular mass index. ^*^P < 0.05, ^**^P < 0.01, significantly different from the C group. ^▲^*P* < 0.05, ^▲▲^*P* < 0.01, significantly different from the Ca group.

After treatment for 8 weeks, LVMI in the WKY group, the Ca group, and the Bh group were significantly lowered when compared to the C group (*P* < 0.05). However, no significant difference between the Ca group and Bh group was identified (Fig. 1).

### Histological characteristics

**Figure 2:**
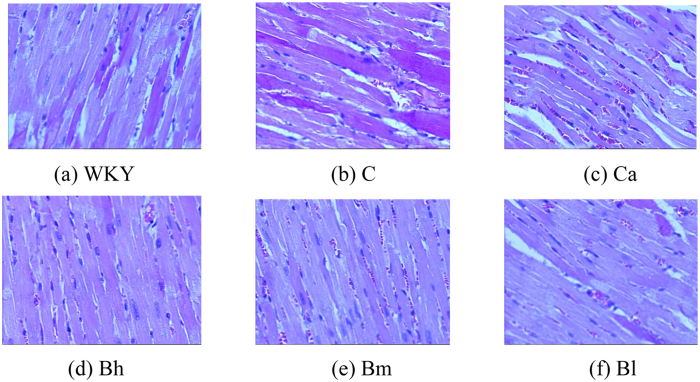
Microscopic observations (200×) of myocardial tissue. After 8 weeks treatment of Bu-Shen-Jiang-Ya decoction, captopril, and distilled water administration, the left ventricular myocardium from 6 groups including (**a**) the WKY group, (**b**) the C group, (**c**) the Ca group, (**d**) the Bh group, (**e**) the Bm group, and (**f**) the Bl group was examined by haematoxylin and eosin.

Eight weeks after the treatment, the cardiac structure was measured through histological examinations. A significant difference was identified in the left ventricular apical biopsy between the C group and the Ca group (Fig. 2, HE staining 200x magnification). The single ventricular myocytes in the Bh group and the Ca group were larger than those in the C group.

### Effect of BSJYD on the proteins expression of ERK signaling by WB analysis

**Figure 3:**
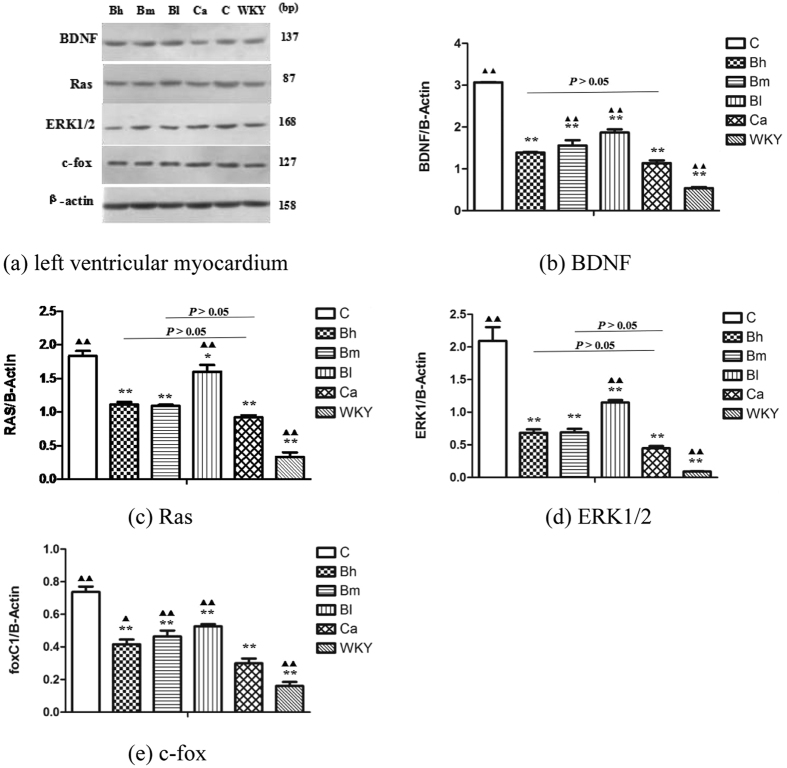
Effect of Bu-Shen-Jiang-Ya decoction on the proteins expression levels of ERK signaling in the left ventricular areas by western blotting analysis. (**a**) The expression levels of BDNF, Ras, ERK1/2, and c-fox in the left ventricular myocardium of the 6 groups. (**b**) The expression level of BDNF of the 6 groups in the left ventricular myocardium. (**c**) The expression level of Ras of the 6 groups in the left ventricular myocardium. (**d**) The expression level of ERK1/2 of the 6 groups in the left ventricular myocardium. (**e**) The expression level of c-fox of the 6 groups in the left ventricular myocardium. Detailed information about the protein expression of ERK signaling was shown in the Supplementary Information file. ^*^P < 0.05, ^**^P < 0.01, significantly different from the C group. ^▲^*P* < 0.05, ^▲▲^*P* < 0.01, significantly different from the Ca group.

Western blotting analysis was performed to examine the proteins expression of ERK signaling pathway, including BDNF, Ras, ERK1/2, and c-fox in left ventricular myocardium among the six groups (n = 12 per group). Figure 3 (a) showed the expression of these proteins in the left ventricular areas. As shown in Fig. 3(b), the expression of BDNF was significantly increased in the C group, the Ca group, the Bh group, the Bm group, and the Bl group, when compared with that of the WKY group (*P* < 0.01). However, it was significantly decreased in the Bh group and the Ca group when compared with the C group (*P* < 0.01). However, no significant difference between the Ca group and Bh group on the expression of BDNF was identified (*P* > 0.05). As shown in Fig. 3(c), the expression of Ras was significantly increased in the C group, the Ca group, the Bh group, the Bm group, and the Bl group when compared with the WKY group (*P* < 0.01). However, when compared with that of the C group, the expression level of Ras was reduced in the Ca group, the Bh group, the Bm group, and the Bl group (*P* < 0.05). As compared to the Ca group, Ras was increased in the Bl group (*P* < 0.05); but no statistically significant differences were observed in the Bh group and the Bm group in terms of their expression of Ras (*P* > 0.05). As shown in Fig. 3(d), the expression of ERK1/2 was significantly reduced in the Ca group, the Bh group, the Bm group, the Bl group, and the WKY group when compared to the C group (*P* < 0.01). As compared to the Ca group, it was significantly increased in the Bl group (*P* < 0.05); however, no statistically significant difference was identified in the Bh group and the Bm group (*P* > 0.05). As shown in Fig. 3(e), c-fox was significantly increased in the C group, the Ca group, the Bh group, the Bm group, and the Bl group compared with the WKY group (*P* < 0.05). However, the expression of c-fox was decreased in the Ca group, the Bh group, the Bm group, the Bl group, and the WKY group when compared with the C group (*P* < 0.05). It was increased in the Bh group (*P* < 0.05), significantly increased in the Bm group and the Bl group compared to the Ca group (*P* < 0.01).

### Effect of BSJYD on the mRNA expression of ERK signaling by Real-time PCR assay

**Figure 4:**
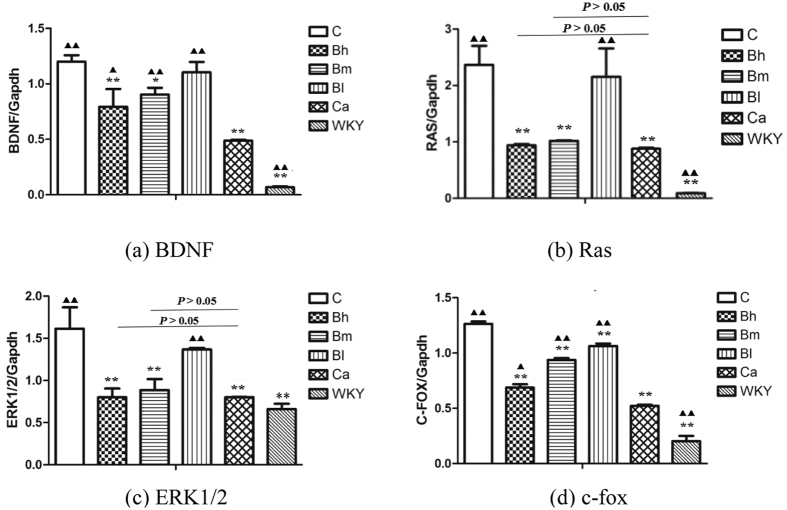
Effect of Bu-Shen-Jiang-Ya decoction on the mRNA expression levels of ERK signaling in the left ventricular areas by Real-time PCR assay. (**a**) The expression level of BDNF mRNA of the 6 groups in the left ventricular myocardium. (**b**) The expression level of Ras mRNA of the 6 groups in the left ventricular myocardium. (**c**) The expression level of ERK1/2 mRNA of the 6 groups in the left ventricular myocardium. (**d**) The expression level of c-fox mRNA of the 6 groups in the left ventricular myocardium. ^*^P < 0.05, ^**^P < 0.01, significantly different from the C group. ^▲^*P* < 0.05, ^▲▲^*P* < 0.01, significantly different from the Ca group.

Real-time PCR was also performed to examine the mRNA expression of BDNF, Ras, ERK1/2, and c-fox in left ventricular myocardium. As shown in Fig. 4(a), the mRNA expression of BDNF in the C group, the Ca group, the Bh group, the Bm group, and the Bl group were significantly increased when compared to the WKY group (*P* < 0.01). As compared with the C group, the expression of BDNF mRNA in the Ca group, the Bh group, and the Bm group were significantly decreased (*P* < 0.01), while there is no difference in the Bl group (*P* > 0.05). As compared with the Ca group, the expression of BDNF mRNA in the Bh group, the Bm group, and the Bl group were significantly increased (*P* < 0.05). As shown in Fig. 4(b), the mRNA expression of Ras in the C group, the Bh group, the Bm group, the Bl group, and the Ca group were significantly increased when compared to the WKY group (*P* < 0.05). As compared with the C group, the expression of Ras mRNA in the Ca group, the Bh group, and the Bm group were significantly decreased (*P* < 0.01), while there is no difference in the Bl group (*P* > 0.05). As compared with the Ca group, the expression of Ras mRNA in the Bl group was significantly increased (*P* < 0.01), while there were no difference in the Bh group and the Bm group (*P* > 0.05). As shown in Fig. 4(c), the mRNA expression of ERK1/2 in the C group, the Bh group, the Bm group, and the Bl group were significantly increased (*P* < 0.01); while there is no difference in the Ca group when compared to the WKY group (*P* > 0.05). The expression of ERK1/2 mRNA in the Ca group, the Bh group, and the Bm group were significantly decreased (*P* < 0.01), while there is no difference in the Bl group (*P* > 0.05) when compared to the C group. As compared to the Ca group, the expression of ERK1/2 mRNA in the Bl group was significantly increased (*P* < 0.01), while there were no difference in the Bh group and the Bm group (*P* > 0.05). As shown in Fig. 4(d), the mRNA expression of c-fox in the C group, the Ca group, the Bh group, the Bm group, and the Bl group were significantly increased when compared to the WKY group (*P* < 0.01). The expression of c-fox mRNA in the Ca group, the Bh group, the Bm group, and the Bl group were significantly decreased when compared with the C group (*P* < 0.01). As compared with the Ca group, c-fox mRNA in the Bh group, the Bm group, and the Bl group were significantly increased (*P* < 0.05).

## Discussion

Hypertension could be classified into the categories of “vertigo” and “headache” in TCM theory^28^. Pattern or syndrome is the basic unit and key concept of TCM^29^. It has been identified that TCM patterns are associated with clinical manifestations of hypertension and its related target organ damage^30^. According to the diagnosis method of syndrome differentiation, kidney yin deficiency syndrome is the critical type of hypertension, which included dizziness, headache, tinnitus, dysuria, weakness, fatigue, sexual dysfunction, lassitude in loins and legs, red tongue with less fur, and deep thready pulse^31^,^32^. A literature analysis of 13, 272 hypertensive patients revealed that kidney yin deficiency syndrome account for about 26.27% and the proportion of kidney yin deficiency syndrome increased with age^33^,^34^. Therefore, Chinese herbal medicine nourishing kidney yin deficiency have been widely utilized by TCM physicians in the treatment of hypertension, especially in elderly patients^35^. Recent researches have demonstrated that traditional Chinese medicinal herbs and formulas with kidney yin-tonifying effect display good clinical antihypertensive efficacy and its underlying multiple cardiovascular protective mechanisms might be relevant to inhibiting the activity of sympathetic nerve, blocking the renin-angiotensin system, improving endothelial function, preventing target organ damage, inhibiting inflammation, and improving insulin resistance as well as glucose and lipid metabolism^23^,^36^,^37^. According to the TCM basic theory, BSJYD, which is originated from a famous TCM classic herbal formula Liu Wei Dihuang pill, belongs to the category of Chinese herbal medicine with nourishing kidney yin deficiency effects.

Antihypertensive therapy is the cornerstone of the treatment of hypertension^38^. Clinical trials have confirmed that antihypertensive therapy reduces the risk of cardiovascular disease outcomes, including incident stroke (by 35 to 40%), myocardial infarction (by 15 to 25%), and heart failure (by up to 64%)^39^,^40^,^41^. A recently published SPRINT study demonstrated that an intensive SBP control (less than 120 mmHg) might result in lower rates of fatal and nonfatal major cardiovascular events and death from any cause than standard SBP control (less than 140 mmHg)^42^. That is, strict BP control contributes to more cardiovascular protective effects. In our experiment, a significant reduction of SBP by BSJYD was identified when compared to the C group, and no significant difference between the Bh and C group was identified. Although difference between SHRs and hypertensive patients existed, the variation of BP in SHRs may partially reflect the antihypertensive effect of BSJYD. Biochemically, the pharmacological mechanism of BSJYD is closely related to the major active compounds including eucommia ulmoides lignans, gastrodin, polysaccharides of gastrodia rhizome, panax notoginseng saponins, and paeonol. Previously published studies also supported the BP-lowering effect of Chinese herbal medicine with nourishing kidney yin deficiency effects. A systematic review involving 6 randomized controlled trials with 527 people demonstrated that, compared with antihypertensive drugs alone, Chinese herbal formula with kidney-tonifying effects as adjunctive therapy significantly lowered SBP by 8.69 mmHg in patients with senile hypertension^43^. Another systematic review including 6 randomized trials and a total of 555 hypertensive participants revealed a significant reduction of 9.31 mmHg of SBP and 6.27 mmHg of DBP by Liu Wei Dihuang pill as complementary therapy when compared to antihypertensive drugs alone^44^. Thus, this study provided evidence that BSJYD could be used to lower BP and is beneficial for hypertension treatment.

Considerable evidence suggesting the important role of autonomic nervous system in BP regulation and development of hypertension have been established^45^,^46^,^47^. HR is regarded as an independent risk factor for hypertension^48^. A clinic HR (more than 79 bpm) is a significant predictor of all-cause, cardiovascular, and noncardiovascular mortality in untreated older patients with isolated systolic hypertension^49^. The elevated resting HR should be paid more attention to in hypertensive patients^50^. In this experiment, HR in the Bh group was significantly decreased, suggesting that BSJYD is beneficial in inhibiting sympathetic nervous activity, thus lowering HR in SHRs. Biochemically, some active ingredients from the components of BSJYD, including hawthorn extract and paeonol, have a certain effect in reducing HR. Previous studies identified that oral administration of paeonol result in a reduction of 20.25 mmHg in SBP and HR simultaneously^51^. Therefore, BSJYD not only exhibited certain antihypertensive effects, but also lowered HR significantly, which is similar to the pharmacological mechanism of β-blocker.

The results of our study showed a dose-dependently suppression of cardiac hypertrophy remodeling effect by BSJYD. It has been recognized that, in the chronic hypertension, elevated BP contributes to wall thickening and functional changes chronically in response to specific conditions, including LVH and cardiac fibrosis^52^. Increased LVM or LVH is a frequent complication of arterial hypertension, which is also an indicator of end-organ damage. The presence of LVH is associated with an increased rate of cardiovascular morbidity and mortality independent of other cardiovascular risk factors and, notably, independent of BP values^53^. Interestingly, both BP reduction and LVH suppression were accompanied by favorable outcome in the present research. It was identified that SHR continued to develop further high BP level, which could also be seen in other studies on SHR model, leading to a higher incidence of LVH when compared with WKY rats^54^,^55^. Obvious formation of cardiac hypertrophy and over expression of EKR signaling pathway were identified in the C group. In our experimental study, after administration of BSJYD with 8 weeks, we were able to demonstrate marked reduction of LVMI, improvement in histological examination, and down regulation of EKR signaling pathway to some degree in the SHRs treated with BSJYD compared with untreated animals. It was suggested that the suppression effect of BSJYD on LVH may relate to the down-regulation of EKR signaling pathway.

In summary, the SHR treated with a daily dose of BSJYD over a period of 8 weeks demonstrated not only reduction in BP and HR with a dose-dependently manner, but also protection of target organ damage in hypertension. In this respect, our findings were consistent with previous reports about Chinese herbal medicines on SHR model^56^,^57^,^58^,^59^,^60^. We can draw the following conclusions from the present study: (a) BSJYD treatment could decrease BP and HR, and suppress LVH remodeling to a certain extent in SHR; (b) BSJYD treatment can inhibit the expression of BDNF, Ras, ERK1/2, and c-fox mRNA in LVH; and (c) a dose-dependent cardiovascular protective effects of BSJYD was identified.

## Conclusions

Our present study suggested that BSJYD and captopril inhibit cardiac hypertrophy *via* a mechanism that may involve the regulation of the EKR signaling pathway. BSJYD treatment decreased BP and HR efficiently and reversed ventricular remodeling of SHRs. The mechanisms were possibly associated with the suppressive effect of BSJYD on the EKR signaling pathway. Therefore, our findings provided a theoretical basis for using BSJYD in the treatment of hypertension and its associated myocardial hypertrophy and fibrosis. BSJYD may be a new candidate cardioprotective drug for patients with hypertensive vascular diseases, which should be given priority for future preclinical and clinical studies.

## Materials and Methods

### Composition of BSJYD

**Figure 5:**
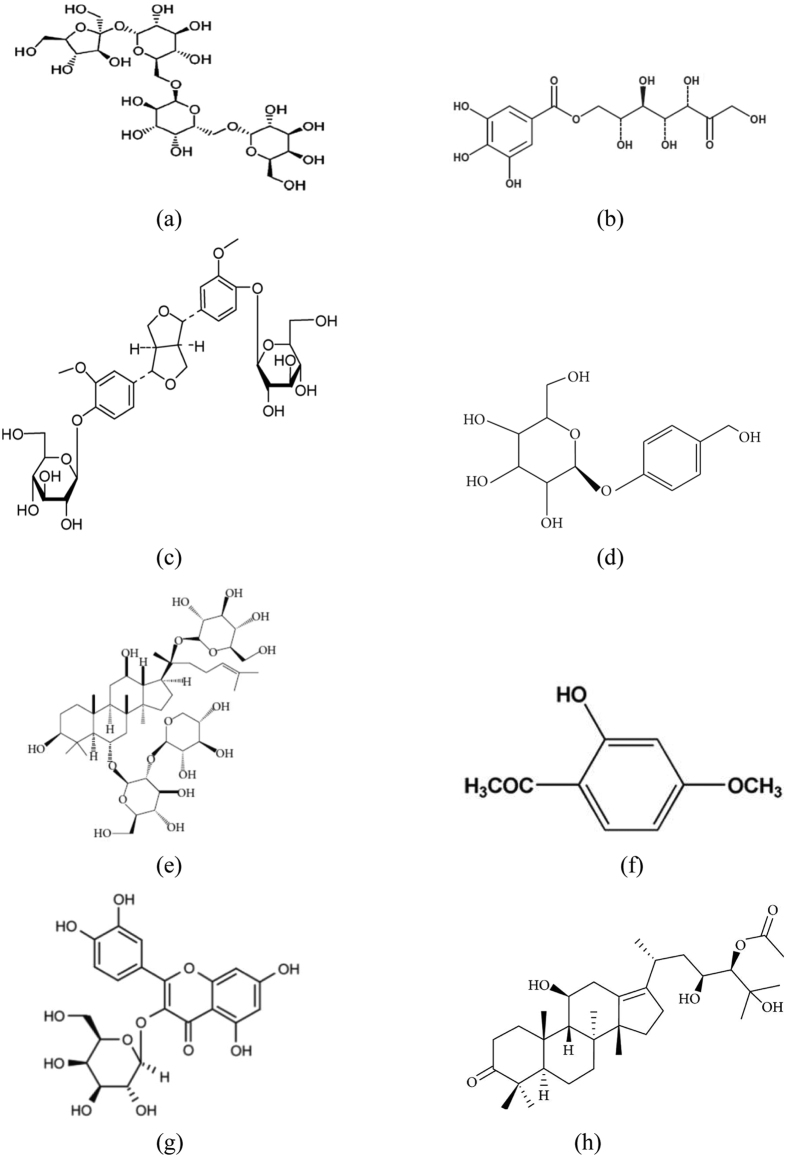
Chemical structure of the main active ingredients of Bu-Shen-Jiang-Ya decoction. (**a**) Stachyose of Rehmannia Glutinosa oligosaccharide. (**b**) 7-O-galloyl-D-sedoheptulose of Corni Fructus. (**c**) (+)-pinoresinol-di-β-d-glucopyranoside of Eucommia Bark. (**d**) Gastrodin of Gastrodia. (**e**) Ginsenoside R1 of Notoginseng Root. (**f**) Paeonol of Cortex of the Peony Tree Rote. (**g**) Hyperoside of Crataegus Fruit. (**h**) Alisol A of Alisma.

BSJYD consists of 8 commonly used Chinese herbs as shown in Table 3. The main chemical components of BSJYD were described in Fig. 5. All of these herbs with granule forms were provided and identified by Neo-Green Pharmaceutical Co., Ltd (*Sichuan*, China). The powdered BSJYD compounds, stored at 4 °C, were dissolved in distilled water prior to use.

### Experimental animals

All animal experimental methods were carried out in accordance to the guidelines of China legislations on the ethical use and care of laboratory animals. The study protocol was approved by the Animal Review Board of the Guang’anmen Hospital, China Academy of Chinese Medical Sciences. Sixty 12-week-old male SHRs, weighing 240–280 g, and twelve 12-week-old male Wistar-Kyoto (WKY) rats, weighing 260–300 g, were purchased from Beijing Vital River Laboratory Animal Technology Co., Ltd. with certificate number: SCXK (*Beijing*) 2012-0001. Each cage contained one rat which had free access to laboratory chow and water and with the normal circadian rhythm. Ambient temperatures were between 18 °C and 22 °C with humidity 45–70%.

### Administration of drugs

Sixty SHRs were trained for two weeks and then randomly allocated into five groups of twelve rats each. From the start of the third week, the rats were given drugs at 8:00 am every morning. The doses and routes are as follows: BSJYD high dose group (6.75 g/ml, Bh), BSJYD middle dose group (3.375 g/ml, Bm), BSJYD low dose group (1.6875 g/ml, Bl); 1 group which received captopril (0.125 g/ml, Ca); and 1 control group of untreated SHRs (C). Additionally, a control group of 12 WKY rats was established. Both control groups (C and WKY) were treated with distilled water. The drugs were administered for 8 weeks. All rats used in this study received humane care.

### Measurement of SBP and heart rate (HR)

SBP of all rats was measured using the indirect tailcuff plethysmographic method with a rat tail BP monitor (BP-100A, a noninvasive computerized tail-cuff system for measuring blood pressure in mice, Chengdu Technology & Market Co., LTD, *Sichuan*, China). Rats were kept calm and conscious till pulsatory signals from the arteria caudilis were displayed steadily. At least 10 determinations were made on each rat and the mean of 6 readings within a 5–10 mmHg range was taken as the SBP^61^. HR was recorded at the same time. BP and HR were measured every 2 weeks (4 times totally).

### Measurement of left ventricular mass index (LVMI)

After this period, all rats were sacrificed with urethane anesthesia. Their hearts were removed quickly and washed with cold normal saline, then dried with filter paper. Left ventricle was separated, and left ventricular mass (LVM) was accurately weighed by electronic balance. Then, left ventricular mass index (LVMI) was calculated by LVM/body weight ratios. Hearts of all animals were immediately stored in liquid nitrogen until western blot (WB) analysis and Real-time PCR (RT-PCR) were performed.

### Histological examination

Left ventricular myocardium was cut into transverse sections and stained with haematoxylin and eosin (H&E)^62^. The stained sections were examined under a light microscope (OLYMPUS BX51, Japan) and photographed at 200x magnification for morphological analysis.

### Western blot analysis

The left ventricle of each rat was taken, kept at −80 °C, and prepared for WB analysis. The following antibodies were used: BDNF (1: 500, Santa Cruz Biotechnology Inc.), Ras (1: 1000, Santa Cruz Biotechnology Inc.), ERK1/2 (1: 1000, Santa Cruz Biotechnology Inc.), and c-fox (1:1000, Cell Signaling Technology Inc.). Proteins were separated by 8% SDS-PAGE and transferred to PVDF membrane (Millipore, Temecula, CA, USA), which were then incubated with primary antibodies at 4 °C. The membranes were further incubated with horseradish peroxidase-conjugated secondary antibodies (1:10000) for 1 hour at room temperature. ECL visualisation was performed, and the resulting images were captured using the Gene Gnome Gel Imaging System (Syngene Co.). Image J (NIH image, Bethesda, MD, USA) was used to analyze the gel images.

### Real-time PCR

Left ventricle samples were frozen with the use of liquid nitrogen. Total RNA was extracted by use of TRIZol reagent (Invitrogen). Total RNA was quantified by spectrophotometry and reverse transcribed with use of the M-MLV Reverse Transcriptase System (Promega, Madison, WI) with oligo (dT) primers. mRNA expressions of BDNF, Ras, ERK1/2, and c-fox in left ventricle were examined by Real-time PCR with the use of LightCycler (Roche Applied Science, Indianapolis, IN) following the manufacturer’s instructions. Primers used for PCR were as follows: β-actin: upstream, 5′-GACAAGATGGTGAAGGTCG-3′, downstream, 5′-GTGGGTAGAGTCATACTGGAACAT-3′, product: 158 bp; BDNF: upstream, 5′-CCTCCTCCCATTTTGGTCCC-3′, downstream, 5′-CACTGTTTTCCACGAGTGTCA-3′, product: 137 bp; Ras: upstream, 5′-AAGGACCAGTTCCCAGAGGT-3′, downstream, 5′-CCAACTCTACCTGCTTCCCG-3′, product: 87 bp; ERK1/2: upstream, 5′-GGCATCCGAGACATCCTCAG-3′, downstream, 5′-TATGTACTTGAGGCCCCGGA-3′, product: 168 bp; and c-fox: upstream, 5′-GGGAGCTGACAGATACGCTC-3′, downstream, 5′-GGTTCAGCCTTCAGCTCCAT-3′, product: 127 bp. The mRNA sequences were obtained from GenBank (Bethesda, MD). Quantitative values were obtained from the threshold cycle value (Ct), the point at which a significant increase in fluorescence was first detected. Experiments were performed in triplicate for each data point, and the data were analyzed with the 2^−△△Ct^ method^63^. The results of RT-PCR were confirmed by gel electrophoresis.

### Statistical analysis

All experimental data were expressed as the mean ± SD. The data was statistically evaluated using one-way analysis of variance (ANOVA), and a post hoc analysis was performed using Fisher’s least significant difference (LSD) test. The SPSS computer program (version 18.0) was used for data analyses. *p* value less than 0.05 was considered to be statistically significant.

## Additional Information

**How to cite this article:** Xiong, X. *et al*. Traditional Chinese medicine suppresses left ventricular hypertrophy by targeting extracellular signal-regulated kinases signaling pathway in spontaneously hypertensive rats. *Sci. Rep.* **7**, 42965; doi: 10.1038/srep42965 (2017).

**Publisher's note:** Springer Nature remains neutral with regard to jurisdictional claims in published maps and institutional affiliations.

## Figures and Tables

**Table 1:** Effect of Bu-Shen-Jiang-Ya decoction on systolic blood pressure.

Group	n	0 week	4 weeks	8 weeks
WKY	12	120.71 ± 8.79**^▲▲^	125.98 ± 9.08**^▲▲^	126.77 ± 8.54**
C	12	201.73 ± 9.43	212.63 ± 8.16^▲▲^	218.65 ± 9.72^▲▲^
Ca	12	200.66 ± 10.56	164.77 ± 8.52**	170.28 ± 10.12**
Bh	12	202.27 ± 9.06	184.37 ± 10.35**^▲^	178.13 ± 8.76**
Bm	12	201.54 ± 10.06	193.03 ± 9.89^▲^	188.04 ± 7.86**^▲^
Bl	12	200.81 ± 9.32	190.17 ± 8.91^▲^	190.65 ± 9.79**^▲^

^*^P < 0.05, ^**^P < 0.01, significantly different from the C group.

^▲^*P* < 0.05, ^▲▲^*P* < 0.01, significantly different from the Ca group.

**Table 2:** Effect of Bu-Shen-Jiang-Ya decoction on heart rate.

Group	n	0 week	4 weeks	8 weeks
WKY	12	366.74 ± 26.83**^▲▲^	345.91 ± 25.42**^▲▲^	331.77 ± 27.92**
C	12	412.89 ± 19.56	396.26 ± 23.83^▲▲^	370.51 ± 23.91^▲▲^
Ca	12	408.36 ± 20.72	391.88 ± 22.61	361.69 ± 20.07
Bh	12	402.52 ± 24.07	376.63±23.01*^▲^	344.43 ± 18.93**^▲^
Bm	12	399.25 ± 25.80	394.42 ± 22.80	383.74 ± 27.03^▲^
Bl	12	405.48 ± 21.84	391.37 ± 18.02	387.45 ± 23.91^▲▲^

^*^P < 0.05, ^**^P < 0.01, significantly different from the C group.

^▲^*P* < 0.05, ^▲▲^*P* < 0.01, significantly different from the Ca group.

**Table 3:** Composition of Bu-Shen-Jiang-Ya decoction.

English name	Latin name	Chinese name	Place of production (Province)	Collecting time (Season)	Part used	Amount used (g)	TCM efficacy	Main chemical components	Pharmacological activity	Ref
Rehmannia	Radix Rehmanniae Glutinosae	Dihuang	*Henan*	Autumn	Root	25	Nourishing the liver and kidney yin and enriching blood	Rehmannia glutinosa polysaccharide, rehmannia glutinosa oligosaccharides, and rehmannioside	Lowering BP and improving glucose metabolism, lipid metabolism, and insulin resistance	64,65,66,67
Cornus Fruit	Corni Fructus	Shanzhuyu	*Henan*	Autumn	Fruit	10	Nourishing the liver and kidney yin, inducing astringency, and preventing prostration	Iridoid glycoside, aglycone, tannins, polysaccharide, organic acid, and ester	Lowering blood glucose, improving lipid metabolism and insulin resistance, and protecting vascular endothelial cells	68,69,70,71,72,73
Eucommia Bark	Cortex Eucommiae Ulmoidis	Duzhong	*Sichuan*	Summer	Bark	10	Nourishing the liver and kidney, strengthening muscles and bones, and soothing the fetus	Eucommia ulmoides lignans, Eucommia ulmoides iridoids, isoquercitrin, rutin, eucomman A, penylpropannoids, and quercetin	Lowering BP (NO↑, RAAS↓), reversing hypertensive vascular remodeling and hypertensive cardiac remodeling, improving lipid metabolism (HMG-CoA reductase↓, Apo I↑), lowering blood glucose, and improving insulin resistance	74,75,76,77,78,79
Gastrodia	Gastrodiae Rhizoma	Tianma	*Sichuan*	Spring	Tuber	20	Calming the liver, relieving spasm, and subduing wind	Gastrodin and polysaccharides of gastrodia rhizome	Lowering BP (PPARγ↑, RAAS↓), improving lipid metabolism (AMPK↑) and insulin resistance, and impairing vascular endothelial function (ET-1↓, eNOS↑)	80,81,82
Notoginseng Root	Notoginseng Radix	Sanqi	*Yunnan*	Spring	Root	3	Promoting blood circulation to remove blood stasis, stopping bleeding, dispersing swelling, and relieving pain	Panax notoginseng saponins (Ginsenoside Rb1, R1, and Rg1, Notoginsenoside R1, etc.)	Lowering BP (PI3K/Akt/eNOS pathway↑), protecting the vascular endothelium, and improving prethrombotic state and lipid metabolism	83,84,85
Cortex of the Peony Tree Rote	Cortex Radicis Moutan	Mudanpi	*Anhui*	Spring	Root bark	10	Eliminating pathogenic heat, cooling the blood, and promoting blood circulation to remove blood stasis	Paeonol	Lowering BP and heart rate, increasing the arterial blood flow, and improving glucose metabolism (AMPK↑)	86,87,88
Crataegus Fruit	Crataegi Fructus	Shanzha	*Henan*	Autumn	Fruit	30	Promoting digestion, removing food stagnation, expelling tenia, and promoting blood circulation to remove blood stasis	Hyperoside, apigenin, luteolin, quercetin, kaempferol, and herbacetin	Lowering BP and heart rate, improving lipid metabolism,and improving vascular endothelial dysfunction	89,90,91
Alisma	Rhizoma Alismatis	Zexie	*Fujian*	Winter	Rhizome	30	Inducing diuresis, excreting dampness, and clearing heat	Alisol A, alisol B, and alisol A 24-acetate	Improving lipid metabolism and lowering blood glucose (HMG-CoA reductase↓, PPARα↑, leptin↓)	92,93

Abbreviations: ↑: up-regulation; ↓: down-regulation; AMPK: phosphorylated AMP activated protein kinase; Ang II: Angiotensin II; Apo: apolipoprotein; BP: blood pressure; eNOS: endothelial nitric oxide synthase; ET: endothelin; HMG-CoA: 3-hydroxy-3-methyl glutaryl coenzyme A; NO: nitric oxide; PPARγ: peroxisome proliferator-activated receptor γ; RAAS: renin-angiotensin-aldosterone system; TCM: traditional Chinese medicine.
